# Supplementary material for: Evaluation of strategies using simulation model to control a potential outbreak of highly pathogenic avian influenza among poultry farms in Central Luzon, Philippines
Source: PLoS One. 2020 Sep 10;15(9):e0238815. doi: 10.1371/journal.pone.0238815 (PMC7482972; doi:10.1371/journal.pone.0238815)
Supplement: S1 File — (DOCX) [file pone.0238815.s004.docx]

Sensitivity analysis results of Latent period related with HPAI H5N6 spread in Commercial farms in Central Luzon, Philippines. The results are based on 120,000

simulations (1,000 simulations/scenario)

| Parameter | Mean duration  (95% CI) | Mean NOIF^1^  (95% CI) | Mean NOPCF^2^  (95% CI) |
| --- | --- | --- | --- |
| **1-2 days^3^** | 75.5  (55.97 – 95.02) | 22.76  (16.76-28.76) | 169.86  (138.12-201.60) |
| 3 days | 92.73  (69.5 – 115.96) | 21.6  (15.90-27.29) | 176.26  (146.89-205.64) |
| 4 days | 99.3  (70.96 – 127.63) | 18.5  (12.70-24.29) | 153.66  (118.67-188.65) |
| 5 days | 91.83  (63.87- 119.79) | 16.86  (10.79-22.93) | 147.96  (113.17-182.75) |

^1^Number of infected farms,^2^number of pre-emptively culled farms,^3^ parameter used

Sensitivity analysis results of notification period related with HPAI H5N6 spread in Commercial farms in Central Luzon, Philippines. The results are based on 120,000

simulations (1,000 simulations/scenario)

| Parameter | Mean duration  (95% CI) | Mean NOIF^1^  (95% CI) | Mean NOPCF^2^  (95% CI) |
| --- | --- | --- | --- |
| 1 | 33.9  (21.93-45.86) | 8.46  5.69-11.23 | 119.86  93.18-146.55 |
| 2 | 32.03  (26.23- 37.83) | 8.5  6.13-10.86 | 122.66  99.51-145.81 |
| 3 | 37.8  (31.44-44.15) | 12.76  8.57-16.96 | 140.86  111.99-169.73 |
| 4 | 46.93  (37.67-56.19) | 15.46  11.46-19.46 | 153.9  123.37-184.42 |
| **5-7^3^** | 75.5  (55.975- 95.02) | 22.76  16.76-28.76 | 169.86  138.12-201.60 |

^1^Number of infected farms,^2^number of pre-emptively culled farms,^3^ parameter used

Sensitivity analysis results of detection period related with HPAI H5N6 spread in Commercial farms in Central Luzon, Philippines. The results are based on 120,000

simulations (1,000 simulations/scenario)

| Parameter | Mean duration  (95% CI) | Mean NOIF^1^  (95% CI) | Mean NOPCF^2^  (95% CI) |
| --- | --- | --- | --- |
| 3 | 37.8  31.75-43.84 | 12.7  9.48-15.91 | 136.86  111.10-162.62 |
| 4 | 52.2  36.74-67.65 | 15.23  11-19.46 | 150.7  124.74-176.65 |
| **5-7^3^** | 75.5  55.97-95.02 | 22.76  16.76-28.76 | 169.86  138.12-201.60 |

^1^Number of infected farms,^2^number of pre-emptively culled farms,^3^ parameter used

Sensitivity analysis results of spatial kernel constants related with HPAI H5N6 spread in Commercial farms in Central Luzon, Philippines. The results are based on 120,000

simulations (1,000 simulations/scenario)

| Parameter | Mean duration  (95% CI) | Mean NOIF^1^  (95% CI) | Mean NOPCF^2^  (95% CI) |
| --- | --- | --- | --- |
| Current^3^ | 75.5  55.97-95.97 | 22.76  16.76-28.76 | 169.86  138.12-201.60 |
| Boender | 67.83  52.06-83.60 | 28.03  21.87-34.19 | 184.53  158.92-210.14 |
|  |  |  |  |

^1^Number of infected farms,^2^number of pre-emptively culled farms,^3^ parameter used
