# Supplementary material for: Evaluation of strategies using simulation model to control a potential outbreak of highly pathogenic avian influenza among poultry farms in Central Luzon, Philippines
Source: PLoS One. 2020 Sep 10;15(9):e0238815. doi: 10.1371/journal.pone.0238815 (PMC7482972; doi:10.1371/journal.pone.0238815)
Supplement: Attachment — Submitted filename: Specific comments towards PONE-D-20-13166.docx [file pone.0238815.s005.docx]

Specific comments towards **PONE-D-20-13166**:

1. Line 2: “Influenza” —>”influenza”
2. Line 44: “preemptive” is one single word, so please correct the “Pre-emptive” through the whole manuscript.
3. Line 49: “duration” should be more specific, as “pandemic duration”.
4. Line 52: remove “will”.
5. Lie 53: “post detection”, I think you mean “after the detection“.
6. Line 77: remove “all” and “present”.
7. Line 78: I don’t think this is a grammatically correct sentence, please re-construct this sentence.
8. Line 84: Theoretically, culling should always be effective. So, it is “generalizable”. Please re-write this sentence.
9. Line 87: “will”—>”can”.
10. Line 92: “will be”—>”should be”.
11. Line 99: “impact”—>”impacts”.
12. Line 119: “The farm profiles were compiled from farm visits”. You included more than 1,100 farms in this study, did you visited all of them? If you just visited part of these farms, then how did you compile the non-visited farms?
13. Line 137-138: These parameters were generated by MLE, but how and why? Please add more details and explanations.
14. Line 142-166: These mathematical models should just be simply presented here. Please add more details and explanations.
15. Line 193-197: If I understand the manuscript correctly, you randomly chose one farm as index farm, right? Then You totally chose 100 farms through your whole study. So under each of the scenario, you chose 1 farm as index, and run the simulation 1000 iterations, and then repeat this process for 100 times? Please make this part clearer.
16. Table 2: Please explain NPEC more clearly. What do you mean with “No culling with reporting within 24 hours…..”. It is not clear enough.
17. Line 207: Please clearly define “epidemic duration”. Do you mean from the first infection occurring to last infected farm disappeared?
18. Line 262-263: you mentioned the percentage of duck farm, but is it important in your study? I didn’t mentioned farm type can influence anything before.
19. Line 268: “an additional 0.5 km”—>”1.5km”.
20. Line 289-304: You mentioned lots of location names, but they are not necessary because (1) you didn’t label this location name in figure; (2) your simulation can not be interpreted as reality. I think you better to compare the distribution of outbreaks by overlapping them on the same figure. Remember, this is a qualitative not quantitative study. Moreover, using this names can only confuse your readers because they do not know where are the places.
21. Line 372: “The results presented here…”—>”Our results…”
22. Line 381: how do you know that the current control measure is “adequent”? What’s the meaning of your “adequate”? I think this should be explained better, and more careful.
23. Line 388-293: You compared the nr. of culled farms, pandemic duration and nr. of infected farms through the manuscript. But I think a more clearer and direct way to present (or compare) these three result is building a 3D plot. For example, z-axis is duration, x-axis is nr. of infected farms, y-axis is nr. of culled farms. I believe you can find a trade-off effect by making this plot.
24. Line 403-404: I don’t think you can select a “best” control strategy by doing this research, but more likely (or objectively) to show the trade-off effects among these control measure, by comparing their effects on duration, nr. of infected farms and nr. of culled farms. I think discussed about this trade-off is more objective and reasonable than “selecting the best strategy“, and concluding “current strategy is adequent”.
25. Line 408: Likewise, the location names doesn’t mean anything in current context.
26. Line 409: Sometimes you use “BAI”, sometimes use “Bureau of Animal Industry”. Please be consistent.
27. Line 412-416. Please re-organize this part, making it more clear.
28. Line 424. “.” is missing.
29. Line 424-427. You mentioned that “R01 can still be a source of infection”, and then immediately, you also mentioned that culling in farms with such a small R0 brings no benefit. Please re-organize this sentence, making it more clear and concise.
30. Line 430. I think you have forgotten to upload you Fig. 14.
31. Line 435. As Before, location names don’t mean anything here, unless you show them with labels in a figure.
32. Line 436: Again, this is a qualitative not quantitative study. You can compare the effects of various strategies, and discussed the trade-off, but it is far-fetched to make a conclusion about “safe” strategy. How do you define “safe”, and from which aspect you think this strategy is “safe”? Be careful with making a conclusion like this.
33. Line 448-449: Please re-organize this sentence, making it clearer and concise.
34. Line 457-458: I am confused here. I don’t think this can be assigned as “advantage” of NPEC. If the pandemic is not over, but the political decision makes people MISTAKENLY believe the pandemic is over, it may cause more problems. Please be careful with this opinion. I think you should clarify this part.
35. Line 479-480. “Neighbors have a higher risk of becoming infected”. It seems to be a reasonable conclusion in your study, but you can expand the discussion a bit more. Actually, the transmission doesn’t only depend on distance, but also depends on connection. If two farms (one is susceptible, another one is infected) are far from each other, but very well connected by transportation, then the transmission may very much likely happen between them.
36. Line 494: “with”—>”in”.
37. Line497: remove “by Thrusfield (2005)”.
38. Line 501: add example after “Biological pollution is also possible”.
39. Line 502-503: “…more acceptable sociopolitically…”
40. Line 504: You sometimes use “foot-and-mouth disease”, sometimes use “FMD”. Please be consistent.
41. Line 515-516: please rephrase this sentence.
42. Line 518: remove the “.”.
43. Line 524-525: replace “to be… on” with “depends on”.
44. Line 529: What do you mean with the “region wide standard protocol”? Please make it clearer.
45. Line 529-530: It seems there is a transition from your discussion to conclusion. You mentioned the current culling strategy is enough, but you concluded that culling is enough in high risk area, whereas culling is not optimal in low risk area in your conclusion part. The transition is not very well discussed. And you didn’t separate (or define) the high risk area and low risk area. I suggest to sort the ideas, and explain them step by step.
